# RETRACTION: Exploring Wound Management in Dental Pulp: Utilizing Single‐Cell RNA Sequencing for Global Transcriptomic Analysis in Healthy and Inflamed Pulpal Tissues

**DOI:** 10.1111/iwj.70539

**Published:** 2025-04-18

## Abstract

**RETRACTION:** GuN. , WangY. , SunG. , ZouH. , YangN. , SunX. , and LiuZ. , “Exploring Wound Management in Dental Pulp: Utilizing Single‐Cell RNA Sequencing for Global Transcriptomic Analysis in Healthy and Inflamed Pulpal Tissues,” International Wound Journal 21, no. 3 (2024): e14804, 10.1111/iwj.14804.38385817 PMC10883240

The above article, published online on 22 February 2024, in Wiley Online Library (http://onlinelibrary.wiley.com/), has been retracted by agreement between the journal Editor in Chief, Professor Keith Harding; and John Wiley & Sons Ltd. Following an investigation by the publisher, all parties have concluded that this article was accepted solely on the basis of a compromised peer review process. The editors have therefore decided to retract the article. The authors did not respond to our notice regarding the retraction.

**RETRACTION:** N. Gu , Y. Wang , G. Sun , H. Zou , N. Yang , X. Sun , and Z. Liu , “Exploring Wound Management in Dental Pulp: Utilizing Single‐Cell RNA Sequencing for Global Transcriptomic Analysis in Healthy and Inflamed Pulpal Tissues,” International Wound Journal 21, no. 3 (2024): e14804, 10.1111/iwj.14804.38385817 PMC10883240

The above article, published online on 22 February 2024, in Wiley Online Library (http://onlinelibrary.wiley.com/), has been retracted by agreement between the journal Editor in Chief, Professor Keith Harding; and John Wiley & Sons Ltd. Following an investigation by the publisher, all parties have concluded that this article was accepted solely on the basis of a compromised peer review process. The editors have therefore decided to retract the article. The authors did not respond to our notice regarding the retraction.

